# High‐Density Vertical Transistors with Pitch Size Down to 20 nm

**DOI:** 10.1002/advs.202302760

**Published:** 2023-08-08

**Authors:** Zhaojing Xiao, Liting Liu, Yang Chen, Zheyi Lu, Xiaokun Yang, Zhenqi Gong, Wanying Li, Lingan Kong, Shuimei Ding, Zhiwei Li, Donglin Lu, Likuan Ma, Songlong Liu, Xiao Liu, Yuan Liu

**Affiliations:** ^1^ Key Laboratory for Micro‐Nano Optoelectronic Devices of Ministry of Education, School of Physics and Electronics Hunan University Changsha 410082 China

**Keywords:** graphene/Au hybrid drain, pitch size, vertical field effect transistor

## Abstract

Vertical field effect transistors (VFETs) have attracted considerable interest for developing ultra‐scaled devices. In particular, individual VFET can be stacked on top of another and does not consume additional chip footprint beyond what is needed for a single device at the bottom, representing another dimension for high‐density transistors. However, high‐density VFETs with small pitch size are difficult to fabricate and is largely limited by the trade‐offs between drain thickness and its conductivity. Here, a simple approach is reported to scale the drain to sub‐10 nm. By combining 7 nm thick Au with monolayer graphene, the hybrid drain demonstrates metallic behavior with low sheet resistance of ≈100 Ω sq^−1^. By van der Waals laminating the hybrid drain on top of 3 nm thick channel and scaling gate stack, the total VFET pitch size down to 20 nm and demonstrates a higher on‐state current of 730 A cm^−2^. Furthermore, three individual VFETs together are vertically stacked within a vertical distance of 59 nm, representing the record low pitch size for vertical transistors. The method pushes the scaling limit and pitch size limit of VFET, opening up a new pathway for high‐density vertical transistors and integrated circuits.

## Graphical abstract

By combining thin Au film with monolayer graphene, thin hybrid drain electrodes are realized with low sheet resistance, leading to the construction of ultra‐short vertical field effect transistor with pitch size down to 20 nm and on‐state current of 730 A cm^−2^.

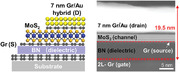

## 1 Introduction

Graphene based vertical field effect transistors (GVFETs) have attracted considerable interest for high performance electronics.^[^ ^1^, ^2^, ^3^, ^4^ ^]^ Within this device structure, the semiconductor channel is vertically sandwiched between metal drain and graphene source electrodes, where the tunable Fermi level of graphene could enable effective control of vertical carrier injection across graphene‐semiconductor junction.^[^ ^4^, ^5^ ^]^ In particular, the channel length (vertical distances between two electrodes) of a GVFET is determined by the semiconductor thickness and can be easily scaled down to sub‐10 nm regime (ref. [5]). More importantly, vertical transistors can be further stacked on top of another through monolithic 3D integration and does not consume additional chip footprint beyond what is needed for a single device at the bottom.^[^ ^6^, ^7^, ^8^ ^]^ Therefore, high‐density transistors are expected in vertical direction without the need of sophisticated high‐resolution lithography, opening up another dimension for high‐density devices and further extending Moore’ Law.

**Figure 1:**
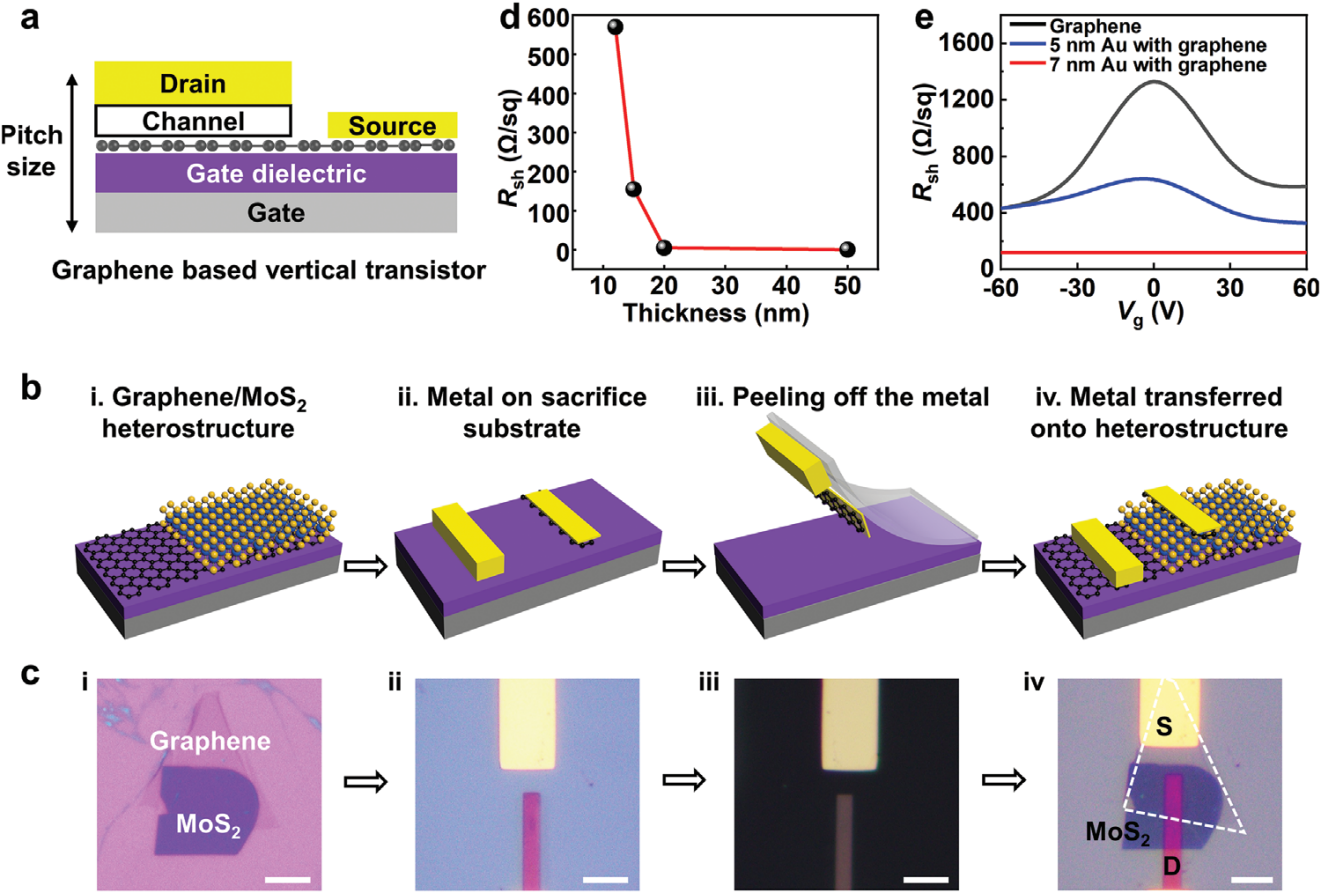
Scaling of drain electrode thickness and the fabrication processes of ultra‐thin GVFET. a) Schematic of the GVFET, where the vertical pitch size is determined by the thickness of all functional layers. b) Fabrication processes of GVFET with ultra‐scaled drain electrode, including four essential steps: i) fabrication of graphene/MoS_2_ heterostructure, ii) drain pre‐fabrication on a sacrificial substrate, iii) metal mechanically peeling‐off, iv) vdW integration of drain on the graphene/MoS_2_ heterostructure. c) Corresponding optical image of each fabrication step, and the scale bars are 3 µm. d) The measured *R*_sh_ of Au film as a function of film thickness. e) The *R*_sh_ of monolayer graphene (black curve), 5 nm thick graphene/Au hybrid film (blue curve), and 7 nm thick graphene/Au hybrid film (red curve), under various gate voltages.

However, fabricating high‐density GVFETs with small pitch size is technologically challenging, and could be largely attributed to the vertical scaling of drain metals. In a typical back‐gated GVFET, five essential layers are subsequently deposited in the vertical direction, including gate layer, dielectric layer, source layer, channel layer, and drain layer, as schematically illustrated in **Figure** **1**a. The total pitch size is simply determined by adding their layer thicknesses, hence, reducing the thickness of each component has been a major focus for GVFET scaling. In recent years, the source layer and channel layer have been scaled to atomic thickness (<1 nm) using monolayer graphene and molybdenum disulfide (MoS_2_), respectively;^[^ ^4^, ^5^, ^9^ ^]^ similarly, the gate dielectric and gate electrode could also be scaled to sub‐5 nm thickness using 2D boron nitride (BN) and graphene.^[^ ^10^, ^11^, ^12^, ^13^, ^14^, ^15^ ^]^ However, the vertical scaling of drain metal remains of great challenge due to the trade‐offs between metal thickness and conductivity.^[^ ^16^, ^17^ ^]^ Reducing the thickness of conventional metal below 20 nm, the resistance of drain electrode could exponentially increase and eventually become discontinuous (open‐circuit) when thickness decreases to sub‐10 nm (ref. [18, 19]). On the other hand, 2D metal (such as graphene) has been applied as the drain electrode for thickness scaling, however, large contact resistance is typically observed due to the limited work function modulation and the limited density of states of graphene electrode.^[^ ^2^, ^11^ ^]^ Therefore, the thinnest metal drain electrode of GVFET is over 20 nm and the pitch size is typically over 100 nm (ref. [5, 20, 21, 22]), greatly limiting the scaling of GVFET.

In this article, we report a simple approach to scale the drain electrode to sub‐10 nm and the total GVFET pitch size down to 20 nm. By combining 7 nm thick Au with monolayer graphene, the hybrid film demonstrates metallic behavior with low sheet resistance (*R*_sh_) of ≈100 Ω sq^−1^, which is over one order of magnitude <7 nm thick Au or monolayer graphene. Furthermore, by van der Waals (vdW) laminating the hybrid drain on top of 3 nm thick MoS_2_, the delicate lattice of vertical short‐channel can be largely retained. Together, we can scale the whole GVFET to a smallest thickness of 20 nm, including all essential component layers, and the device demonstrates a higher on‐state current of 730 A cm^−2^. Furthermore, we vertically stacked three individual GVFETs together within a vertical distance of 59 nm, corresponding to a smallest vertical pitch size of 20 nm, representing the record low pitch size for not only vertical transistors but also conventional planar transistors, to the best of our knowledge. Our method pushes the scaling limit and pitch size limit of GVFET, opening up a new pathway for high‐density vertical transistors and integrated circuits.

## 2 Results and Discussion

### 2.1 GVFET Fabrication Processes with Ultra‐Scaled Drain Electrode

Figure 1b schematically illustrates the device structure and fabrication processes. To fabricate the device, monolayer graphene is first mechanically exfoliated onto a silicon substrate (with 300 nm SiO_2_), working as the bottom source electrode. Next, MoS_2_ flake (3 to 5 nm thick) is mechanically exfoliated as the channel material and dry‐transferred on top of graphene source, as shown in Figure 1b,i. We note the dry transfer process here is essential to obtain a clean graphene/MoS_2_ interface with minimized residues or defects, enhancing the carrier transport in vertical direction. In the meantime, ultra‐thin graphene/Au hybrid film is pre‐fabricated on a sacrificial Si substrate as the drain electrode, by depositing 7 nm thick Au on top of monolayer graphene, as shown in Figure 1b(ii). Finally, metal electrodes are mechanically released and physically laminated on top of graphene/MoS_2_ vertical heterostructures using our previous vdW integration technique,^[^ ^23^ ^]^ as shown in Figure 1b(iii–iv). The corresponding optical images of fabrication steps are shown in Figure 1c, and the details of the vdW integration process are described in Experimental Section.

We note our device structure and vdW lamination processes are unique to achieve ultra‐scaled vertical transistors, owing to two factors. First, in term of the drain thickness, the graphene/Au hybrid film is essential to reduce its thickness to ≈7 nm, overcoming the conductivity limitation of ultra‐thin metal film. To demonstrate this, we have measured the sheet resistance of Au film with various thickness, as well as our graphene/Au hybrid film. As shown in Figure 1d, the *R*_sh_ of Au film increases quickly with thickness reducing below 20 nm, indicating the film surface roughness and scattering start to impact electron transport. With further reducing thickness below 10 nm, Au film is totally non‐conducting due to film discontinuity and Au islands formation, as confirmed through SEM (scanning electron microscopy) image in Figure S1 (Supporting Information). In contrast, for graphene/Au hybrid film with 7.3 nm total thickness (0.3 nm graphene plus 7 nm thick Au), metallic behavior and *R*_sh_ of ≈100 Ω sq^−1^ could still be observed (red curve in Figure 1e) due to the better wettability of the Au film on graphene substrate (SEM image in Figure S1, Supporting Information), which is low enough to maintain the electrical properties of GVFET. Furthermore, the measured *R*_sh_ is much lower than that of monolayer graphene (black curve in Figure 1e), and importantly, the hybrid film does not show any gate modulation behavior, indicating the semi‐metallic behavior of graphene disappears and the film acts as pure conductor.^[^ ^24^ ^]^ In contrast, by further reducing the Au thickness from 7 to 5 nm, the hybrid film (5 nm Au with monolayer graphene) shows much increased *R*_sh_ and a clear Dirac point, as highlighted by the blue curve in Figure 1e suggesting the hybrid film is semi‐metallic. Therefore, we choose 7 nm thick Au with graphene as the optimized thickness for drain electrode scaling.

Second, besides the scaling of drain electrode, our vdW lamination process is also essential for the scaling of channel length of GVFET. As demonstrated in previous studies, directly depositing metals on the ultra‐thin vertical channel is highly destructive, since the deposition process usually involves repeated bombardment to the channel region with high energy hot atoms or atomic clusters, leading to considerable interface damage, defects, and surface states.^[^ ^23^, ^25^, ^26^ ^]^ The disordered contact interface is particularly critical for ultra‐thin GVFET where the contact region is essentially the whole channel, leading to vertical leakage current paths and eventually short‐circuit.^[^ ^5^ ^]^ Therefore, short vertical channels (<10 nm) can hardly be fabricated using the direct evaporation process, limiting the scaling of vertical channel length. In contrast, within our process, the hybrid drain film is pre‐fabricated on a sacrificial wafer and then physically laminated on top of the ultra‐thin MoS_2_ channel, where the delicate vertical channel is not impacted by the harsh fabrication process. Hence, ultra‐scaled channel length could be realized with thickness of 3 nm, which is also important to reduce the total pitch size.

### 2.2 Electrical Measurement of GVFET with 7 nm Thick Drain Electrode

**Figure 2:**
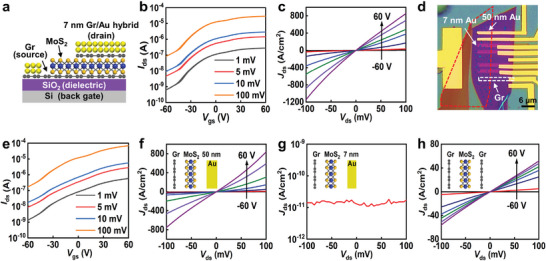
Electrical measurement of GVFETs with different drain electrodes. a) Cross‐section schematic of our GVFET with 7 nm graphene/Au hybrid drain electrode. b) *I*_ds_−*V*_gs_ transfer characteristics of GVFET with 7 nm graphene/Au hybrid drain electrode at various bias voltage of 1, 5, 10, and 100 mV. c) *I*_ds_−*V*_ds_ output characteristics of GVFET with 7 nm graphene/Au hybrid drain electrode under various gate voltages from −60 to 60 V (20 V step). d) Optical image of GVFETs with different drain electrodes, including 50 nm thick Au, 7 nm thick Au, and monolayer graphene. The bottom monolayer graphene source electrode is highlighted by a red box, top monolayer graphene drain electrode is highlighted by a white box. e) *I*_ds_−*V*_gs_ transfer characteristics of GVFET with 50 nm thick Au drain electrode. f–h) Electrical measurement of GVFETs with various drain electrodes of f) 50 nm thick Au, g) 7 nm thick Au, and h) monolayer graphene, and their corresponding device schematics are shown as the insets.

Electrical transport studies of the resulting devices were carried out at room temperature in a probe station under vacuum condition (1.2 × 10^−5^ Torr). As shown in **Figure** **2**a, the bottom graphene is always grounded as the source electrode, the top graphene/Au hybrid electrode is biased as the drain electrode and the back‐gate dielectric is 300 nm thick SiO_2_. Figure 2b,c shows the *I*_ds_–*V*_gs_ transfer curve and *I*_ds_–*V*_ds_ output curve of the resulting device. In general, the current density increases with increasing positive gate potential, demonstrating that the electrons are the majority charge carriers in vertical transistor, consistent with the n‐type MoS_2_ (ref. [5, 8, 21]). The on‐off ratio of the short‐channel device is over 320 (Figure 2b), also consistent with previous GVFETs with sub‐3 nm channel length,^[^ ^5^ ^]^ and could be further enhanced by increasing the channel length (2D thickness) of the device.^[^ ^5^ ^]^ In addition, the short channel advantage of vertical transistors allows the overall electrical properties is dominated by the vertical carrier transport rather than planar transport, consistent with previous literatures.^[^ ^8^ ^]^ The current density could be further calculated by normalizing the total current with vertical overlapping area (between hybrid drain and graphene source), resulting in a highest current density of 850 A cm^−2^ at 0.1 V bias and 6000 A cm^−2^ at 0.5 V bias, as shown in Figure 2c and Figure S2 (Supporting Information). It is particularly important to note that this current density is over 3 orders of magnitude larger than the vertical tunneling transistors^[^ ^11^ ^]^ and barristors^[^ ^9^ ^]^ at the same *V*_ds_ bias, which may also be largely attributed to our ultra‐short channel length.

To further evaluate the vertical transistor performance using 7 nm graphene/Au hybrid drain, we have pre‐fabricated three different drain electrodes and then vdW integrating them on the same bottom graphene/MoS_2_ stack as the control samples. As shown in Figure 2d, the first drain electrode is 50 nm thick Au, the second one is 7 nm thick Au, and the third one is monolayer graphene. Importantly, these parallel transistors have same bottom electrode and channel flake (fabricated on the same graphene/MoS_2_ heterostructure), and the only difference is the top electrode, providing fair comparison between the top electrode with the device performance. First, for devices with 50 nm thick Au electrode, the device demonstrates an on‐off ratio of 400 and an on‐state current of 837 A cm^−2^ (Figure 2e,f), similar to our device with ultrathin hybrid drain, indicating the 7 nm thick graphene/Au hybrid drain is conducting enough and the electrode resistance won't impact the overall carrier transport. Second, for device with 7 nm thick Au drain electrode, the vertical transistor is totally open‐circuit without any detectable current level (only noise level <10^−10^ A, as shown in Figure 2g). This behavior is expected since film discontinuity and islands formation have been observed for 7 nm Au film (Figure S1, Supporting Information). Third, for device with monolayer graphene as drain electrode, the GVFET demonstrates a small on‐state current of 60 A cm^−2^ at 100 mV bias, which is over one order of magnitude lower than our device with hybrid electrode (Figure 2h). The much‐reduced driving current is not only limited by the larger lateral resistance of graphene electrode, but more limited by the lower carrier density of graphene compared to that of hybrid metal film, since the carrier injection or tunneling possibility is directly related to density states of graphene electrode (between graphene and MoS_2_ channel), hence much reduced carrier injection efficiency. This poor graphene contact at drain side is intrinsically different from bottom graphene/MoS_2_ contact at source side, which can be electrically modulated by the gate voltage and hence more controllable barrier height and injection efficiency.^[^ ^27^ ^]^ In summary, the electrical comparison of three control samples clearly demonstrates our 7 nm thick hybrid drain could simultaneously ensure the high device performance and the scaled drain thickness.

### 2.3 Realization of Ultra‐Scaled GVFET with Thickness Below 20 nm

**Figure 3:**
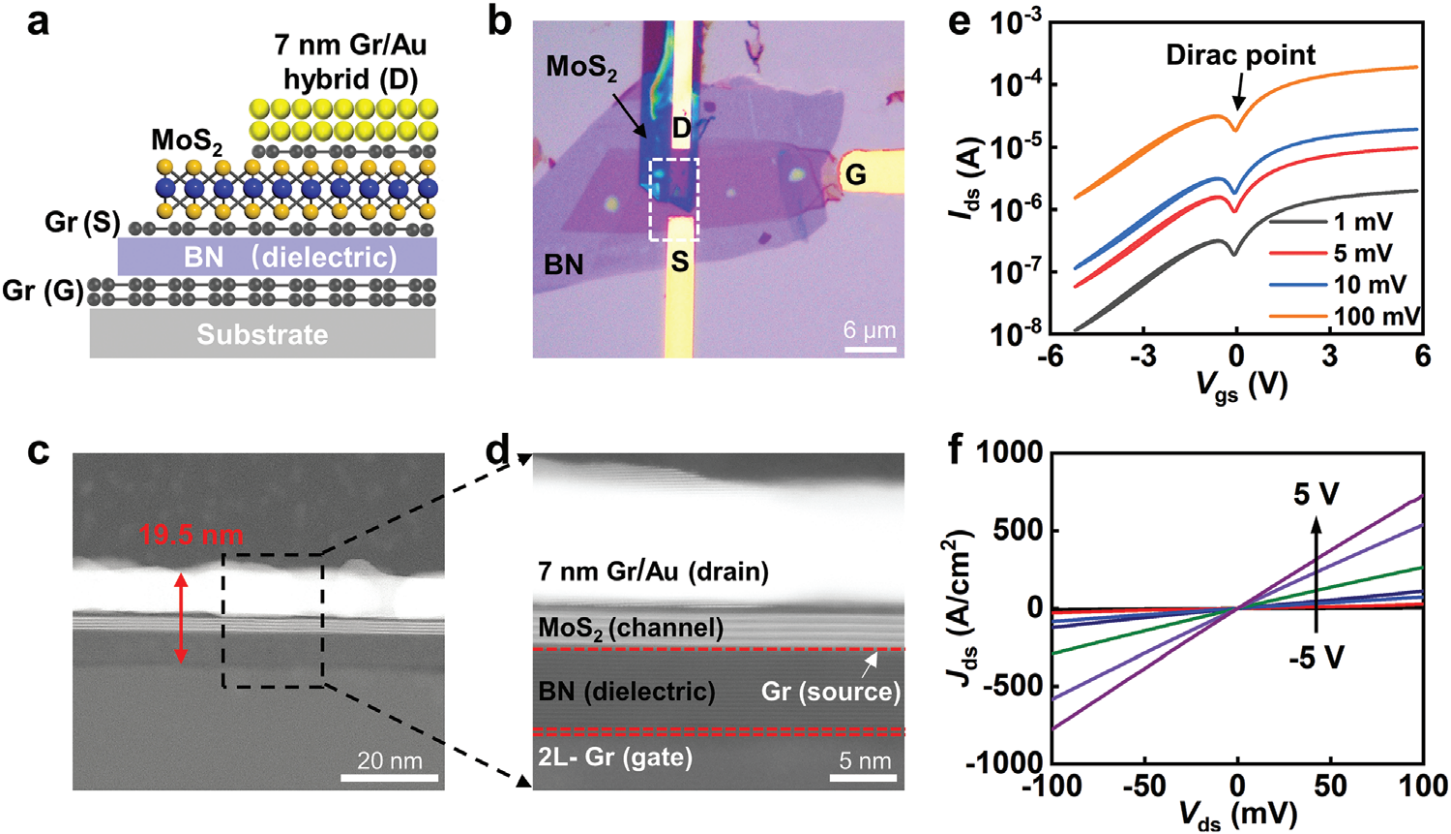
Ultra‐scaled GVFET with total thickness below 20 nm. a,b) Cross‐section schematic and optical image of an ultra‐scaled GVFET, including all essential device layers of gate electrode, dielectric, source, drain, and channel. c,d) High‐resolution transmission electron micrograph (TEM) image of the device, demonstrating the atomic flat interfaces and a total device length of 19.5 nm. Graphene is highlighted by dash line. e) *I*_ds_–*V*_gs_ transfer characteristics of the ultra‐scaled GVFET at various bias voltage of 1, 5, 10, and 100 mV. f) *I*_ds_–*V*_ds_ output characteristics of the ultra‐scaled GVFET at various gate voltages.

With the ability to reduce the hybrid drain thickness to 7 nm, we could construct an ultra‐scaled GVFET to probe its scaling limit. To fabricate this device, bilayer graphene (0.7 nm thick) is used as the gate electrode, 6 nm thick BN is used as the gate dielectric, monolayer graphene (0.3 nm thick) is used as the source electrode, four layers MoS_2_ (2.4 nm thick) is used as the channel, and 7 nm graphene/Au hybrid electrode is used as the drain electrode. These active components are layer‐by‐layer stacked using vdW integration technique, where the device schematics and corresponding optical image are shown in **Figure** **3**a,b and detailed in Figure S3 (Supporting Information). Figure 3c shows the cross‐sectional transmission electron microscope (TEM) image of the corresponding device, and the total device thickness is measured to be 19.5 nm. Importantly, atomic clean interfaces between each layer are observed and could be largely attributed to our vdW stacking process with minimized residues and defects, which is important to ensure the ultra‐thin vertical channel without short‐circuit between source‐drain electrodes, as shown in Figure 3d.

Electrical properties of the resulting device are measured at room temperature within a vacuum probe‐station (1.2 × 10^−5^ Torr). Figure 3e shows the *I*_ds_–*V*_gs_ transfer characteristics of 20 nm thick GVFET under various bias voltage, demonstrating an average on‐off ratio of 160. We note the on‐off ratio is relatively small due to the ultra‐short MoS_2_ channel used here and could be increased over 10^3^ by using thicker MoS_2_ (ref. [5]). Within reducing channel length to such short regime (≈2.4 nm), direct tunneling current starts to emerge between the source‐drain electrode, leading to uncontrollable off‐state leakage current hence reduced on‐off ratio (ref. [5]). Therefore, trade‐offs are needed between the channel length and on‐off ratios, similar as the short channel effect in conventional planar transistors.^[^ ^28^ ^]^ In addition, we note a clear Dirac point is observed in the transfer curve (as highlighted in Figure 3e), which could be attributed to two mechanisms. First, the ultra‐flat surface of BN significantly reduces the electron–hole charge fluctuations, leading to lower substrate doping with clear Dirac point. Furthermore, the channel material MoS_2_ is thinner compared to the device in Figure 2b, resulting in lower channel resistance. Consequently, the lateral resistance of graphene electrode contributes a larger proportion to the total resistance of the device, leading to more visible Dirac point. Figure 3f shows the *I*_ds_‐*V*_ds_ output characteristics of the GVFET, exhibiting a highest current density of 730 A cm^−2^ at a bias voltage of 0.1 V, consistent with the results in Figure 2c using global back gate.

### 2.4 3D Integration of GVFETs with Pitch Size of 20 nm

**Figure 4:**
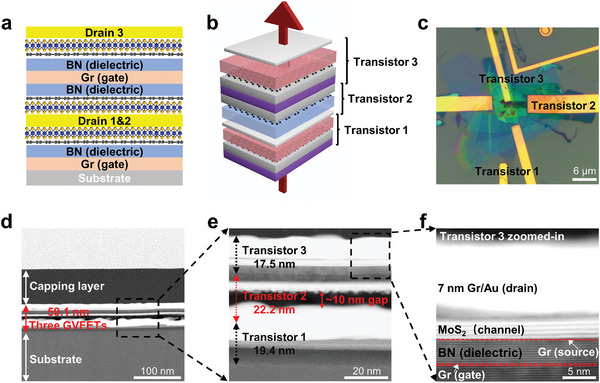
3D Integration for realizing high‐density GVFETs. a,b) Cross‐sectional and perspective schematics of three vertically integrated GVFETs. c) Optical image of the vertically stacked device with three GVFETs. d–f) Corresponding cross‐sectional transmission electron micrograph (TEM) image of three stacked GVFETs with a total length of 59.1 nm. Graphene is highlighted by red dash line.

Achieving high integration density is one of the primary motivations for GVFET scaling, where multiple devices could be stacked together in vertical direction and do not consume additional chip footprint beyond what is needed for a single device placed at the bottom.^[^ ^4^, ^6^, ^8^ ^]^ To demonstrate this, we have fabricated a proof‐of‐concept device by stacking three pre‐fabricated GVFETs in the vertical direction. To save the vertical space, the middle GVFET shares the same drain electrode with the bottom GVFET and the same gate electrode with the top GVFET, as schematically illustrated in **Figure** **4**a,b and detailed in Figure S4 (Supporting Information). The optical image of the final device is shown in Figure 4c and the corresponding cross‐sectional TEM image of our fabricated device is shown in Figure 4d–f. The pitch size of three GVFETs from bottom to top are 19.4, 22.2, and 17.5 nm, respectively, and the total length of three transistors is measured as 59.1 nm (Figure 4d,e), corresponding to an integration density of 5 × 10^4^/mm in vertical direction and a smallest pitch size of 20 nm in average. We note that there is a gap of ≈10 nm between bottom GVFET (transistor 1) and middle GVEFT (transistor 2), which could be attributed to the annealing process during the electrode transfer or could be attributed to the strain induced during the FIB cutting process. At the same time, atomic clean interfaces are observed for the stacked GVFETs with minimized interfacial residues and defects, as shown in Figure 4f. To further validate whether the transfer process impacts electrical performance of our stacked GVFETs, we conducted a comparative analysis by testing the electrical characteristics of the same device before and after transfer process. As shown in Figure S5 (Supporting Information), the *I*_ds_–*V*_gs_ transfer characteristics remain identical by transferring the device to a different substrate, indicating our vdW lamination process will not alter the intrinsic properties of stacked GVFETs. Furthermore, we note although pre‐mature, such small pitch size is indeed a record low value as far as we know, for not only vertical transistors but also planar transistors. The achievement of such small pitch size is the result of the unique structure of VFET, where the size of all essential components is only determined by the layer thickness. In contrast, for conventional planar transistors, the lateral dimension of each component is eventually limited by process resolutions of lithography, etching, or implantation process, where the achievement of 20 nm lateral size for a single pattern (such as channel length, contact length or gate length) is very challenging and the total transistor pitch size could be much larger.^[^ ^29^, ^30^ ^]^

## 3 Conclusion

In summary, we have reported a simple approach to scale the drain electrode to sub‐10 nm and the total GVFET pitch size down to 20 nm. By combining 7 nm thick Au with monolayer graphene, the hybrid drain electrode demonstrates metallic behavior and low *R*_sh_ ≈100 Ω sq^−1^, over one order of magnitude lower than the device with 7 nm thick Au or monolayer graphene as the drain. Importantly, by vdW laminating the hybrid film on top of 3 nm thick MoS_2_, the delicate lattice of ultra‐short vertical channel can be largely retained. Furthermore, we scale all essential component layers to construct an ultra‐scaled GVFET, including gate metal, gate dielectric, source, drain, and vertical channel. The ultra‐scaled GVFET shows a smallest thickness of 19.5 nm as well as high on‐state current of 730 A cm^−2^, consistent with device using thicker drain electrodes. Finally, we stack three individual GVFETs together within a vertical distance of 59 nm, corresponding to a highest integration density of 5 × 10^4^/mm in the vertical direction and a smallest pitch size of 20 nm on average.

## 4 Experimental Section

### Fabrication and Integration Process of the Hybrid Drain Electrode

Monolayer graphene was first mechanically exfoliated on a sacrificial Si wafer and then was etched into a graphene stripe (2 × 10 µm^2^) using oxygen plasma following electron beam lithography. Metallization was performed by thermal evaporation of 7 nm thick Au on top of it to construct a graphene/Au hybrid electrode. At the same time, 50 nm thick Au electrodes were also fabricated on a sacrificial Si wafer by e‐beam lithography followed by thermal deposition. The sacrificial wafer was then functionalized by a hexamethyldisilazane layer in a sealed chamber at 80 °C for 40–60 min and then spin‐coated with poly(methylmethacrylate) (PMMA). After functionalization, the PMMA layer and the metal electrodes (or hybrid electrode) wrapped underneath can be mechanically released using tapes or a thick transparent polydimethylsiloxane stamp owing to the weak adhesion force between PMMA and the functionalized silicon wafer. Next, the metal electrodes could be mechanically released and vdW laminated on the desired target substrate within an ambient atmosphere under an optical microscope. Finally, all pads of the device were exposed by e‐beam lithography for subsequent electrical testing.

## Conflict of Interest

The authors declare no conflict of interests.

## Author Contributions

Y.L. conceived the research. Y.L. and Z.X. designed the experiments. Z.X. performed the device fabrication, electrical measurement, and data analysis. L.K. contributed to the TEM measurement. L.L., Y.C., and Z.L. contributed to device fabrication. L.L., Y.C., X.Y., Z.G., W.L., S.D., D.L., L.M., Z.L., S.L., and X.L. assisted with the electrical characteristic measurements and data analysis. Y.L. and Z.X. co‐wrote the manuscript. All authors discussed the results and commented on the manuscript.

## Supporting information

Supporting Information

## Data Availability

The data that support the findings of this study are available from the corresponding author upon reasonable request.

## References

[1] J. Heo , K. E. Byun , J. Lee , H. J. Chung , S. Jeon , S. Park , S. Hwang , Nano Lett. 2013, 13, 5967. 24256403 10.1021/nl403142v

[2] L. Britnell , R. V. Gorbachev , R. Jalil , B. D. Belle , F. Schedin , A. Mishchenko , T. Georgiou , M. I. Katsnelson , L. Eaves , S. V. Morozov , N. M. Peres , J. Leist , A. K. Geim , K. S. Novoselov , L. A. Ponomarenko , Science 2012, 335, 947. 22300848 10.1126/science.1218461

[3] S. Yuan , Z. Yang , C. Xie , F. Yan , J. Dai , S. P. Lau , H. L. Chan , J. Hao , Adv. Mater. 2016, 28, 10048. 27690190 10.1002/adma.201601489

[4] L. Liu , Y. Liu , X. Duan , Sci. China Inf. Sci. 2020, 63, 201401.

[5] L. Liu , L. Kong , Q. Li , C. He , L. Ren , Q. Tao , X. Yang , J. Lin , B. Zhao , Z. Li , Y. Chen , W. Li , W. Song , Z. Lu , G. Li , S. Li , X. Duan , A. Pan , L. Liao , Y. Liu , Nat. Electron. 2021, 4, 342.

[6] Y. J. Choi , S. Kim , H. J. Woo , Y. J. Song , Y. Lee , M. S. Kang , J. H. Cho , ACS Nano 2019, 13, 7877. 31245996 10.1021/acsnano.9b02243

[7] J. Goldberger , A. I. Hochbaum , R. Fan , P. J. N. l. Yang , Nano Lett. 2006, 6, 973.

[8] W. J. Yu , Z. Li , H. Zhou , Y. Chen , Y. Wang , Y. Huang , X. Duan , Nat. Mater. 2013, 12, 246. 23241535 10.1038/nmat3518 PMC4249642

[9] Y. Liu , G. Zhang , H. Zhou , Z. Li , R. Cheng , Y. Xu , V. Gambin , Y. Huang , X. Duan , Nano Lett. 2017, 17, 1448. 28165746 10.1021/acs.nanolett.6b04417

[10] J. K. Park , S. M. Song , J. H. Mun , B. J. Cho , Nano Lett. 2011, 11, 5383. 22059809 10.1021/nl202983x

[11] Z. Bai , Y. Xiao , Q. Luo , M. Li , G. Peng , Z. Zhu , F. Luo , M. Zhu , S. Qin , K. Novoselov , ACS Nano 2022, 16, 7880. 35506523 10.1021/acsnano.2c00536

[12] L. Britnell , R. V. Gorbachev , R. Jalil , B. D. Belle , F. Schedin , M. I. Katsnelson , L. Eaves , S. V. Morozov , A. S. Mayorov , N. M. Peres , A. H. Neto , J. Leist , A. K. Geim , L. A. Ponomarenko , K. S. Novoselov , Nano Lett. 2012, 12, 1707. 22380756 10.1021/nl3002205

[13] S. Das , R. Gulotty , A. V. Sumant , A. Roelofs , Nano Lett. 2014, 14, 2861. 24754722 10.1021/nl5009037

[14] T. Knobloch , Y. Y. Illarionov , F. Ducry , C. Schleich , S. Wachter , K. Watanabe , T. Taniguchi , T. Mueller , M. Waltl , M. Lanza , M. I. Vexler , M. Luisier , T. Grasser , Nat. Electron. 2021, 4, 98.

[15] F. Wu , H. Tian , Y. Shen , Z. Hou , J. Ren , G. Gou , Y. Sun , Y. Yang , T. L. Ren , Nature 2022, 603, 259. 35264756 10.1038/s41586-021-04323-3

[16] G. Kästle , H. G. Boyen , A. Schröder , A. Plettl , P. Ziemann , Phys. Rev. B 2004, 70, 165414.

[17] R. C. Jaklevic , J. Lambe , Phys. Rev. B 1975, 12, 4146.

[18] J. M. Camacho , A. I. Oliva , Thin Solid Films 2006, 515, 1881.

[19] Y. G. Bi , Y. F. Liu , X. L. Zhang , D. Yin , W. Q. Wang , J. Feng , H. B. Sun , Adv. Opt. Mater. 2019, 7, 1800778.

[20] Y. Sata , R. Moriya , T. Yamaguchi , Y. Inoue , S. Morikawa , N. Yabuki , S. Masubuchi , T. Machida , Jpn. J. Appl. Phys. 2015, 54, 04DJ04.

[21] R. Moriya , T. Yamaguchi , Y. Inoue , S. Morikawa , Y. Sata , S. Masubuchi , T. Machida , Appl. Phys. Lett. 2014, 105, 083119.

[22] W. Li , L. Liu , Q. Tao , Y. Chen , Z. Lu , L. Kong , W. Dang , W. Zhang , Z. Li , Q. Li , J. Tang , L. Ren , W. Song , X. Duan , C. Ma , Y. Xiang , L. Liao , Y. Liu , Nano Lett. 2022, 22, 4429. 35616710 10.1021/acs.nanolett.2c00922

[23] Y. Liu , J. Guo , E. Zhu , L. Liao , S. J. Lee , M. Ding , I. Shakir , V. Gambin , Y. Huang , X. Duan , Nature 2018, 557, 696. 29769729 10.1038/s41586-018-0129-8

[24] K. Nagashio , T. Moriyama , R. Ifuku , T. Yamashita , T. Nishimura , A. Toriumi , Int*. Electron* Devices Meeting, IEEE, Piscataway, NJ 2011.

[25] Y. Liu , Y. Huang , X. Duan , Nature 2019, 567, 323. 30894723 10.1038/s41586-019-1013-x

[26] Y. Jung , M. S. Choi , A. Nipane , A. Borah , B. Kim , A. Zangiabadi , T. Taniguchi , K. Watanabe , W. J. Yoo , J. Hone , J. T. Teherani , Nat. Electron. 2019, 2, 187.

[27] R. Tung , Appl. Phys. Rev. 2014, 1, 011304.

[28] L. Xie , M. Liao , S. Wang , H. Yu , L. Du , J. Tang , J. Zhao , J. Zhang , P. Chen , X. Lu , G. Wang , G. Xie , R. Yang , D. Shi , G. Zhang , Adv. Mater. 2017, 29, 1702522. 10.1002/adma.201702522 28752671

[29] Q. Cao , J. Tersoff , D. B. Farmer , Y. Zhu , S. J. Han , Science 2017, 356, 1369. 28663497 10.1126/science.aan2476

[30] M. Ieong , B. Doris , J. Kedzierski , K. Rim , M. Yang , Science 2004, 306, 2057. 15604400 10.1126/science.1100731

