# Supplementary material for: High‐Density Vertical Transistors with Pitch Size Down to 20 nm
Source: Adv Sci (Weinh). 2023 Aug 8;10(29):2302760. doi: 10.1002/advs.202302760 (PMC10582445; doi:10.1002/advs.202302760)
Supplement: Supplementary file 1 — Supporting Information [file ADVS-10-2302760-s001.pdf]

ADVANCED SCIENCE
Open Access

Supporting Information

for *Adv. Sci.*, DOI 10.1002/advs.202302760

High-Density Vertical Transistors with Pitch Size Down to 20 nm

*Zhaojing Xiao, Liting Liu, Yang Chen, Zheyi Lu, Xiaokun Yang, Zhenqi Gong, Wanying Li, Lingan Kong, Shuimei Ding, Zhiwei Li, Donglin Lu, Likuan Ma, Songlong Liu, Xiao Liu and Yuan Liu**

Supporting Information

High-density vertical transistors with pitch size down to 20 nm

*Zhaojing Xiao, Liting Liu, Yang Chen, Zheyi Lu, Xiaokun Yang, Zhenqi Gong, Wanying Li, Lingan Kong, Shuimei Ding, Zhiwei Li, Donglin Lu, Likuan Ma, Songlong Liu, Xiao Liu, Yuan Liu**

Key Laboratory for Micro-Nano Optoelectronic Devices of Ministry of Education, School of Physics and Electronics, Hunan University, Changsha 410082, China.

*E-mail: yuanliuhnu@hnu.edu.cn

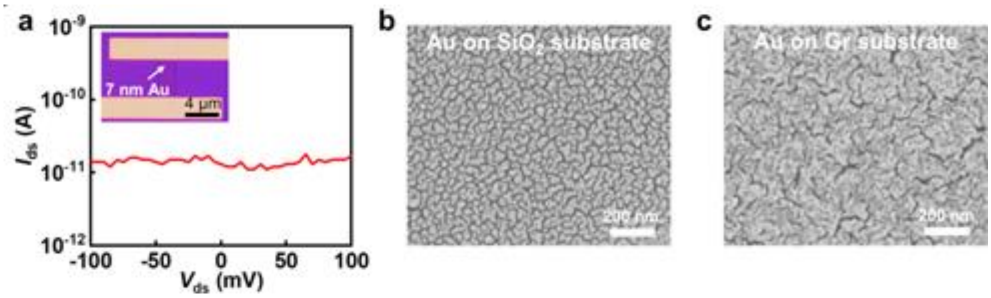

Figure S1. The electrical measurement and SEM (scanning electron microscopy) images of 7 nm Au film. (a) The electrical measurement of 7 nm Au film, with noise current level, indicating the Au film is non-conducting. (b) The SEM image of the 7 nm Au film, exhibiting Au islands formation. (c) The SEM image of the 7 nm graphene/Au hybrid film, exhibiting more continuous Au film formed on graphene substrate.

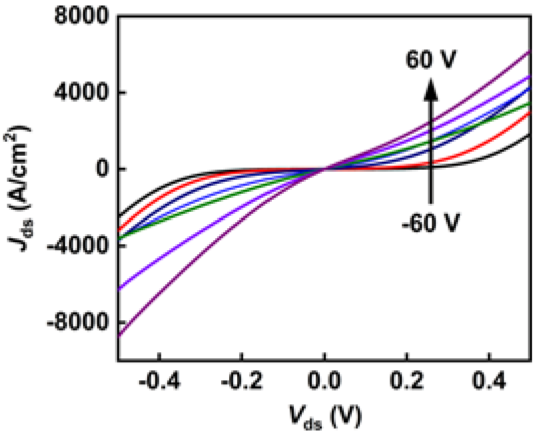

Figure S2. Output curve of ultra-scaled GVFET with a 3 nm thick MoS_2 channel. The highest current density could exceed 6000A cm^{-2} at 0.5 V of bias.

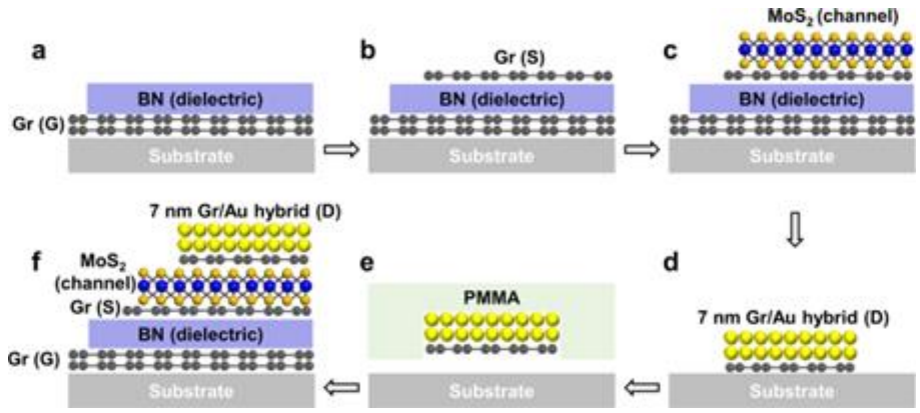

Figure S3. Fabrication processes of ultra-scaled GVFET with 6 steps, including the stacking of bilayer graphene gate and BN dielectric (a), Stacking of monolayer graphene source (b), stacking of few-layer MoS_2 channel (c), pre-fabrication of hybrid drain electrode (d), physical releasing of the hybrid drain (e), vdW lamination of drain electrode (f).

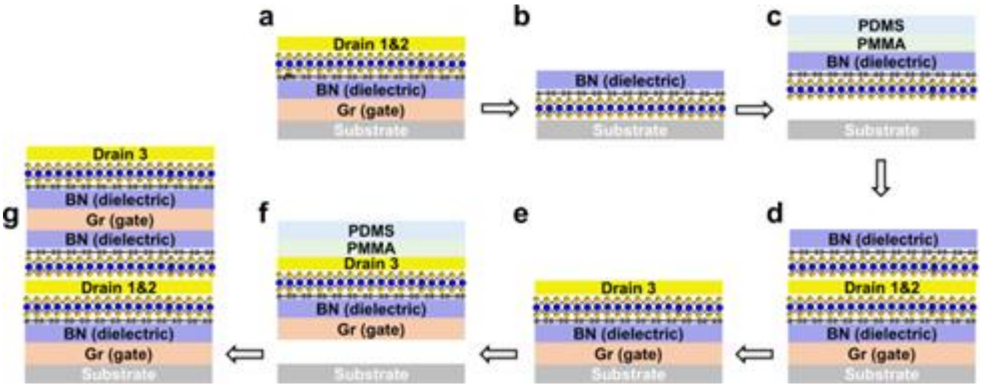

Figure S4. Schematical illustration of stacking processes of three GVFETs, including pre-fabrication of the bottom transistor (a), stacking of middle transistor (b-d), and the integration of top transistor (e-g).

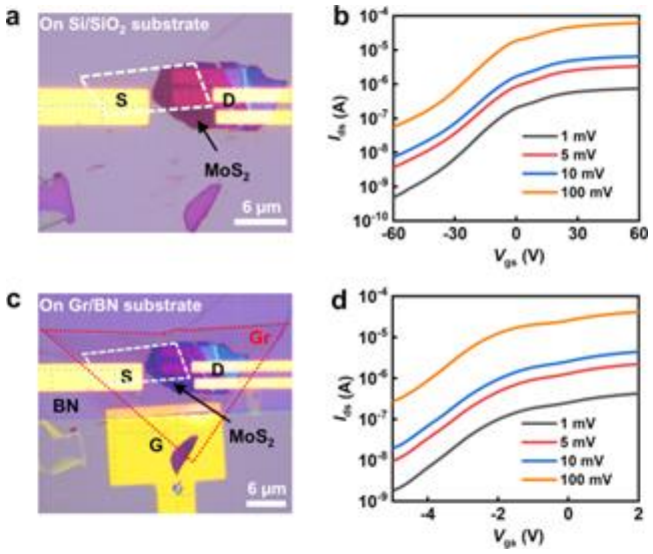

Figure S5. (a,b) Optical image and I_{ds}–V_{gs} transfer characteristics of the GVFET on Si/SiO_2 substrate before transfer. (c,d) Optical image and I_{ds}–V_{gs} transfer characteristics of the GVFET on Gr/BN substrate after transfer, demonstrating similar electrical properties.
